# Selecting Adequate Exposure Biomarkers of Diisononyl and Diisodecyl Phthalates: Data from the 2005–2006 National Health and Nutrition Examination Survey

**DOI:** 10.1289/ehp.1002316

**Published:** 2010-09-22

**Authors:** Antonia M. Calafat, Lee-Yang Wong, Manori J. Silva, Ella Samandar, James L. Preau, Lily T. Jia, Larry L. Needham

**Affiliations:** Division of Laboratory Sciences, National Center for Environmental Health, Centers for Disease Control and Prevention, Atlanta, Georgia, USA

**Keywords:** biomonitoring, DIDP, DINP, exposure, human, NHANES, urine

## Abstract

**Background:**

High-molecular-weight phthalates, such as diisononyl phthalate (DINP) and diisodecyl phthalate (DIDP), are used primarily as polyvinyl chloride plasticizers.

**Objectives:**

We assessed exposure to DINP and DIDP in a representative sample of persons ≥ 6 years of age in the U.S. general population from the 2005–2006 National Health and Nutrition Examination Survey (NHANES).

**Methods:**

We analyzed 2,548 urine samples by using online solid-phase extraction coupled to isotope dilution high-performance liquid chromatography–tandem mass spectrometry.

**Results:**

We detected monocarboxyisooctyl phthalate (MCOP), a metabolite of DINP, and monocarboxyisononyl phthalate (MCNP), a metabolite of DIDP, in 95.2% and 89.9% of the samples, respectively. We detected monoisononyl phthalate (MNP), a minor metabolite of DINP, much less frequently (12.9%) and at concentration ranges (> 0.8 μg/L–148.1 μg/L) much lower than MCOP (> 0.7 μg/L– 4,961 μg/L). Adjusted geometric mean concentrations of MCOP and MCNP were significantly higher (*p* < 0.01) among children than among adolescents and adults.

**Conclusions:**

The general U.S. population, including children, was exposed to DINP and DIDP. In previous NHANES cycles, the occurrence of human exposure to DINP by using MNP as the sole urinary biomarker has been underestimated, thus illustrating the importance of selecting the most adequate biomarkers for exposure assessment.

Phthalates are high-production-volume chemicals. Some phthalates are used as solvents and additives and are often found in personal care products, such as cosmetics, lotions, and perfumes, and in the coatings of some medications (David et al. 2001; Koch and Calafat 2009; Schettler 2006). Others, including diisononyl phthalate (DINP) and diisodecyl phthalate (DIDP), are used primarily as plasticizers of polyvinyl chloride (PVC) and may be used in PVC flooring, wall coverings, building materials, heat-resistant electrical cords, car interiors, and toys (David et al. 2001; Koch and Calafat 2009; Schettler 2006).

Historically, toxicologic studies of phthalates have focused on liver effects in rodents and the toxicologic relevance of these effects to humans (European Commission 2003a, 2003b). Recently, attention has been given to the potential for some phthalates, including DINP, to affect reproductive outcomes and the development of the male reproductive tract (Adamsson et al. 2009; Borch et al. 2006; Gray and Gangolli 1986; Gray et al. 2000; Howdeshell et al. 2007; Kavlock et al. 2002b; Masutomi et al. 2003; Waterman et al. 2000); these phthalates can alter sexual differentiation of the male rat by inhibiting fetal testicular testosterone synthesis (Foster 2006; Gray et al. 2000; Lee et al. 2004; Sharpe and Irvine 2004; Tyl et al. 2004). Other phthalates, such as DIDP, can also produce rodent liver effects (European Commission 2003a) but have not been shown to affect reproductive outcomes (Hushka et al. 2001; Kavlock et al. 2002a). Compared with the large body of experimental evidence suggesting reproductive or developmental toxicity of phthalates, human data are rather limited (Hauser and Calafat 2005; Meeker et al. 2009). Nevertheless, in light of concerns about the potential adverse health effects of phthalates in humans, the European Union has banned several phthalates from cosmetics [National Research Council (NRC) 2008], and several countries, including the United States, have legislation restricting the use of certain phthalates in some children’s products, such as toys. The Consumer Product Safety Commission (CPSC) is currently evaluating whether to lift or make permanent the temporary restrictions, which became effective in February 2009, on the sale of children’s toys that can be placed in a child’s mouth and on child care articles that contain > 0.1% DINP, DIDP or di-n-octyl phthalate (CPSC 2009).

After exposure, phthalates are rapidly metabolized to their corresponding hydrolytic monoesters, which can be further transformed to hydrophilic oxidative products, conjugated, and eliminated (Koch and Calafat 2009). For the past 2 decades, urinary concentrations of these phthalate metabolites have been measured as part of biomonitoring programs or epidemiologic studies, mainly in the United States and Germany [Angerer et al. 2006; Becker et al. 2009; Centers for Disease Control and Prevention (CDC) 2009] and, to a lesser extent, in other countries, to assess exposure of the general population to phthalates.

Biomonitoring data have consistently shown widespread human exposure to multiple phthalates (Becker et al. 2009; CDC 2009; Koch and Calafat 2009; NRC 2008). By contrast, for other phthalates, particularly high-molecular-weight phthalates such as DINP, the exposure appears to be rather limited, based on the concentrations of the corresponding hydrolytic monoesters [e.g., monoisononyl phthalate (MNP)] (CDC 2009; Silva et al. 2004). However, other research suggests that the low frequency of detection of MNP, a metabolite of DINP, in human populations may be attributable partly to the fact that MNP is an insensitive biomarker for DINP exposure assessment because it further metabolizes to form oxidative metabolites before being excreted in urine (Koch and Angerer 2007; Koch et al. 2007). In addition, MNP can be formed from DINP in the environment, but for DINP, as for other phthalates, no environmental sources of oxidative metabolites, including monocarboxyisooctyl phthalate (MCOP), are known (Staples et al. 1997). Based on urinary concentrations of the oxidative metabolites of DINP in 102 German subjects between 6 and 80 years of age, the estimated median intake of DINP was 0.6 μg/kg/day (Wittassek and Angerer 2008); a previous estimate of < 1 μg/kg/day was based on the urinary concentrations of the less-sensitive biomarker of exposure to DINP, MNP (McKee et al. 2004). Toxicokinetics data in animals and in humans suggest that the oxidative metabolites are much more sensitive biomarkers of exposure to DINP than is MNP (Koch et al. 2007; Silva et al. 2006b). Similarly, although only limited DIDP human exposure assessment data exist to date (Silva et al. 2007a), based on animal data (Kato et al. 2007), oxidative metabolites are also likely to be sensitive biomarkers of exposure to DIDP.

Assessing human exposure to phthalates is of interest because of their potential adverse health effects, particularly among the young. Data for several phthalates are available, but exposure information for high-molecular-weight phthalates, including DINP and DIDP, is inadequate (i.e., based on the urinary concentrations of the hydrolytic monoesters) or rather limited (i.e., restricted to adults only). The aims of this study are 3-fold. First, we present for the first time nationally representative data on the concentrations of oxidative metabolites of DINP and DIDP in urine among those people in the U.S. general population ≥ 6 years of age, stratified by age group, sex, and race/ethnicity. Second, we evaluate the exposure to DINP and DIDP, based on the urinary concentrations of their metabolites MCOP and monocarboxyisononyl phthalate (MCNP), respectively, according to select sociodemographic factors. Finally, we also discuss the validity of MNP as a biomarker of exposure to DINP and compare its sensitivity with that of MCOP.

## Materials and Methods

Since 1999, the CDC has conducted the National Health and Nutrition Examination Survey (NHANES) annually. NHANES provides data, released in 2-year intervals, to evaluate the health and nutritional status of the civilian, noninstitutionalized U.S. population of all ages. NHANES includes household interviews, standardized physical examinations, and collection of medical histories and biological specimens. Some of these specimens are used to assess exposure to environmental chemicals.

For this study, we analyzed 2,548 spot urine specimens collected during one of three daily examination sessions from a one-third subset of 2005–2006 NHANES participants ≥ 6 years of age. The representative design of the survey was maintained, because the subset was a random sample of the total NHANES population. The National Center for Health Statistics (NCHS) Institutional Review Board reviewed and approved the study protocol. All participants gave informed, written consent; parents or guardians provided consent for participants < 18 years of age.

The urine samples were shipped on dry ice to the National Center for Environmental Health at the CDC and stored at −20°C or below until analyzed. The analytical method for measuring 15 phthalate monoesters, including MNP and oxidized metabolites of DINPs (MCOP) and DIDPs (MCNP), in 100 μL urine has been described in detail elsewhere (Silva et al. 2007b). The analytical approach involved enzymatic hydrolysis of the conjugated species of phthalate metabolites, followed by online solid-phase extraction, separation with high-performance liquid chromatography, and detection by isotope-dilution negative ion electrospray ionization tandem mass spectrometry. We used the calibration curves constructed with mono(2,6-dimethyl-6-carboxyhexyl) phthalate (for MCOP), mono(2,7-dimethyl-7-carboxyheptyl) phthalate (for MCNP), and mono(3,5,5-trimethyl-1-hexyl) phthalate (for MNP) and their isotopically labeled analogs as the internal standards for quantification, as described previously (Silva et al. 2007b). Calibration standards, quality control, and reagent blank samples were included in each analytical batch along with the study samples (Silva et al. 2007b).

Under our experimental conditions, MCOP and MCNP, the metabolites of DINP and DIDP, respectively, were not chromatographically resolved, and both MCOP and MCNP eluted separately as broad peaks. For quantification, we integrated the whole area under the cluster of peaks encompassing the various isomers of MCOP and MCNP. The hydroxy- and oxo-oxidative metabolites of DINP (Koch and Angerer 2007; Koch et al. 2007) could not be separated adequately; as a result, we could not estimate their concentrations. The limits of detection (LODs)—calculated as 3S_0_, where S_0_ is the standard deviation as the concentration approaches zero (Taylor 1987)—were 0.8 μg/L (MNP), 0.7 μg/L (MCOP), and 0.6 μg/L (MCNP). We prepared low-concentration (4–9 μg/L) and high-concentration (27–58 μg/L) quality control materials with pooled human urine that was analyzed with standards, reagent blanks, and urine samples. The precision of measurements, expressed as the relative standard deviation of multiple measures, depending on the phthalate metabolite, was 8–10% for low-concentration and 6–10% for high-concentration quality control samples.

We used SAS (version 9.2; SAS Institute Inc., Cary, NC) and SUDAAN (version 10; Research Triangle Institute; Research Triangle Park, NC) to perform statistical analyses. SUDAAN calculates variance estimates that account for the complex, clustered design of NHANES. As recommended by NCHS, we used sample population weights to produce estimates that are representative of the U.S. population. We used the log_10_-transformed urinary metabolite concentrations for the statistical analyses and assigned a value equal to the LOD divided by the square root of 2 (Hornung and Reed 1990) to the concentrations below the LOD.

We stratified age, reported in years at the last birthday, in four groups (6–11 years, 12–19 years, 20–59 years, and ≥ 60 years). On the basis of self-reported data, we categorized race/ethnicity as non-Hispanic black, non-Hispanic white, and Mexican American. Participants not defined by these racial/ethnic categories (*n* = 195) were included only in the total population estimate. For each age, sex, and race/ethnic group, we calculated geometric means (GMs) (if the overall weighted frequency of detection was > 60%) and distribution percentiles for both volume-based (micrograms per liter) and creatinine-corrected concentrations (micrograms per gram creatinine). We also determined weighted Pearson correlations among the creatinine-corrected concentrations (log_10_ transformed) of MCOP, MCNP, and MNP in the 334 samples with detectable concentrations of all three compounds. Statistical significance was set at *p* < 0.05.

We used multiple regression to examine whether several variables [i.e., age group, sex, race/ethnicity, creatinine concentration, household income, and examination session (i.e., morning, afternoon, evening)] were associated with the log_10_-transformed urine concentrations of MCOP and MCNP. On the basis of questionnaire responses, annual household income was available in increments of $5,000 (ranging from < $5,000 to > $75,000). We categorized income as < $20,000, $20,000–$45,000, and > $45,000 to obtain a comparable number of participants per group. For the multiple regression models, we used the variables described previously and all their possible two-way interactions to calculate the adjusted GM concentrations (in micrograms per liter) of MCOP and MCNP. These variables were log_10_ transformed, because the distributions of concentrations of these phthalate metabolites and creatinine were skewed.

To arrive at the final model for each analyte, we used backward elimination with SUDAAN to remove the nonsignificant interactions one at a time. Covariates with nonsignificant main effects were then removed one at a time, and the model was rerun to determine whether the beta coefficients for covariates with significant main effects or interactions changed by > 10%. If any did, we retained the relevant nonsignificant covariate in the model. Once the backward procedure was completed, covariates and interactions between covariates were added back into the model one at a time to determine whether any were significant, in which case they were retained in the final model.

We also constructed a 2 × 2 table to examine the suitability of the urinary concentrations of MNP and MCOP as DINP exposure biomarkers.

## Results

In most of the samples analyzed, we detected MCNP (89.9%). Similarly, we detected MCOP in most samples (95.2%), but we detected MNP much less frequently (12.9%). More important, in 82.4% of participants with detectable concentrations of MCOP, which is a sensitive biomarker of exposure to DINP, MNP, the hydrolytic metabolite of DINP, was undetectable (Table 1). The GM and selected percentile concentrations stratified by age, sex, and race/ethnicity are given in Tables 2 and 3 for MCOP and MCNP, respectively, two metabolites that have not been evaluated previously in NHANES, and in the Supplemental Material for MNP [see Supplemental Material, Tables 1 and 2 (doi:10.1289/ehp.1002316)].

**Figure 1:**
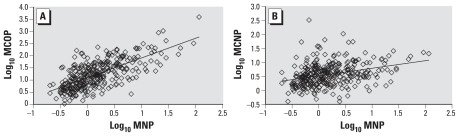
Correlation analyses of the log-transformed creatinine-corrected urinary concentrations of MNP and MCOP (*A*) and of MNP and MCNP (*B*).

Among the 334 persons for whom MNP, MCOP, and MCNP were detectable in the urine, we found statistically significant (*p* < 0.0001) good to moderate correlations between the creatinine-corrected concentrations of MCOP and both MNP [Pearson correlation coefficient (*R*) = 0.63] (Figure 1) and MCNP (*R* = 0.46). We also observed a significant (*p* < 0.0001) but rather weak correlation (*R* = 0.25) between the concentrations of MCNP and MNP (Figure 1). Of interest, the person with the highest concentration of MCOP also had the highest concentration of MNP.

The final MCOP and MCNP models included household income, age group, and log_10_ creatinine without significant interactions between these covariates (Table 4). The log-corrected creatinine concentration increased as the log of the phthalate metabolite concentrations increased [for MCNP, β = 0.82 (95% confidence interval (CI), 0.71–0.94]; for MCOP, β = 0.88 (95% CI, 0.79–0.97)]. See Supplemental Material, Table 3 (doi:10.1289/ehp.1002316). Of interest, the *R*^2^ for the model adjusted for age and income was larger (11% for MCOP; 13% for MCNP) than the *R*^2^ for the model unadjusted for these two variables (see Supplemental Material, Table 3). For both phthalate metabolites, adjusted GM concentrations for children were significantly higher (*p* < 0.01) than for all other age groups; differences between adolescents and adults and between younger and older adults did not reach statistical significance (Table 4). Adjusted GM concentrations for both MCNP and MCOP for persons in the high household income category were significantly higher than for those in the low category (for MCNP, *p* = 0.004; for MCOP, *p* = 0.01). Persons in the medium income level had significantly higher adjusted GM concentrations of MCNP than those in the low-income level (*p* = 0.03); other differences between household income groups did not reach statistical significance (Table 4). If instead of using log_10_ creatinine as a variable in the model, we modeled the log_10_ MCOP and MCNP creatinine-corrected concentrations (in micrograms per gram creatinine), the results were very similar to those presented in Table 4 (shown for MCNP in Supplemental Material, Table 4).

We also conducted weighted univariate and multiple logistic regressions to examine the association of the concentrations of MCOP and MCNP above the 95th percentile (an arbitrary value selected as an example of higher-than-average concentrations) with sex, age group, race/ethnicity, household income, and examination session. We found no covariates significantly associated with the likelihood of MCNP or MCOP exceeding the 95th percentile (data not shown).

## Discussion

We detected MCOP at concentrations ranging from > 0.7 μg/L to 4,961 μg/L in 95.2% of persons examined. By contrast, MNP, the hydrolytic DINP metabolite, was detected in only 12.9% of people and at lower concentration ranges (> 0.8–148.1 μg/L). The only DIDP metabolite evaluated, MCNP, was detected in 89.9% of persons at concentrations of > 0.6–672.6 μg/L. These data suggest that at least 90% of the general U.S. population is exposed to DINP and DIDP. These data are in agreement with previous evaluations of the urinary concentrations of MCOP (Koch et al. 2007; Silva et al. 2006b) and MCNP (Silva et al. 2007a) among select populations of adults in Germany and the United States that suggest that people are exposed to DINP and DIDP. The NHANES 2005–2006 data presented here confirm widespread human exposure to DINP and DIDP and could be used to derive internal dose exposure estimates. Future NHANES data will also be useful to determine the existence of exposure trends. Of note, temporal increases in urinary concentrations of DINP metabolites among some segments of the German population may reflect changes in production and use patterns of DINP between 1988 and 2003 in Germany (Wittassek et al. 2007).

Consistent with expectations, urinary concentrations of MCOP and MNP, which are metabolites of the same parent compound, correlated well with each other. We also found a fair correlation between the urinary concentrations of MCNP and MCOP. Commercial DINP and DIDP formulations are complex mixtures of C8–C10 or C9–C11 phthalates, respectively, enriched in C9 (DINP) or C10 (DIDP) isomeric phthalates (Kavlock et al. 2002a, 2002b). The composition of the mixtures may vary depending on the manufacturing process, but DIDP is likely present in the DINP technical mixtures and vice versa (Kato et al. 2007; Silva et al. 2006a).

Of interest, the person with the highest urinary concentration of MCOP in this study also had the highest urinary concentration of the hydrolytic metabolite of DINP, MNP. These data are in agreement with a previous report on the metabolism and elimination of four DINP metabolites, including MCOP and MNP, in a male adult volunteer after administration of a single oral dose of deuterium-labeled DINP of 1.27 mg/kg body weight (Koch and Angerer 2007). In that individual, most of the DINP recovered in the urine was in the form of oxidative metabolites, including MCOP, and only a very small percentage was in the form of MNP.

We observed that adjusted GM concentrations of MCOP and MCNP were dependent upon age and income. The differences in urinary concentrations of MCOP and MCNP among the various demographic groups examined may reflect differences in the consumption of food or use of consumer products containing DINP and DIDP, respectively. For the first time, we report here the urinary concentrations of some DINP and DIDP metabolites among school-age children and adolescents in the United States. Although we do not have data for toddlers and preschool-age children, our data confirm that exposure to these two phthalates occurs at young ages.

Variability in a person’s exposure to phthalates can result from changes in use of personal care products, diet, or other activities. Although the urinary concentrations of phthalate metabolites can be used to assess a person’s exposure at a single point in time, the predictive ability of one spot sample to categorize exposure over longer time periods will differ among phthalate metabolites; for MCOP and MCNP this temporal variability is unknown. However, several reports suggest that, although some metabolites display more temporal variability than others, the concentrations of several phthalate metabolites in a single urine sample can provide a reliable ranking to classify exposure of an individual to phthalates for up to several months (Adibi et al. 2008; Fromme et al. 2007; Hauser et al. 2004; Hoppin et al. 2002; Peck et al. 2010; Teitelbaum et al. 2008). Furthermore, despite this individual temporal variability, on a population basis (e.g., NHANES), the wide range of concentrations observed likely represents an average exposure scenario (i.e., urinary concentrations in the upper percentiles resulting from the collection of urine soon after a phthalate-related activity may be offset by concentrations in the lower percentiles of other persons who provided the urine specimen shortly before conducting the same activity).

Generating high-quality biomonitoring data requires state-of-the-art analytical chemistry methods as well as controlled sampling protocols and quality control/quality assurance procedures (Angerer et al. 2007; Calafat and Needham 2009; Koch and Calafat 2009; Needham et al. 2005). In addition, the proper interpretation of biomonitoring data requires an understanding of the toxicokinetics of the target compounds (Calafat et al. 2006; Calafat and Needham 2008; Koch and Calafat 2009). For example, a previous DINP exposure assessment by McKee at al. (2004) illustrates the critical importance of absorption, distribution, metabolism, and excretion information. Specifically, these authors concluded that exposure to DINP in the United States was limited (McKee et al. 2004), based on an analysis of NHANES 1999–2000 biomonitoring urinary data (CDC 2009; Silva et al. 2004). However, their estimates were based solely on urinary concentrations of MNP, an insensitive biomarker of environmental exposure to DINP.

Although the frequency of detection and concentration ranges of MNP in NHANES 2005–2006 and NHANES 1999–2000 are quite similar (Silva et al. 2004), NHANES 2005–2006 data for MCOP suggest that > 90% of the U.S. general population is exposed to DINP, a much greater percentage than suspected based on previous NHANES estimates using MNP measurements only. Furthermore, these data also suggest that MNP is a rather insensitive biomarker of background exposures, because it is only a minor metabolic product of DINP in humans (Koch and Angerer 2007; Koch et al. 2007). In contrast, MCOP, a major metabolite of DINP, appears to be a sensitive indicator of DINP exposure. In fact, 82.4% of those classified as exposed to DINP would have been misclassified as unexposed based on urinary concentrations of MNP only. Therefore, we recommend that future biomonitoring studies, particularly those focused on environmental exposures, rely on MCOP or other oxidative metabolites and not solely on MNP.

## Conclusions

We measured the urinary concentrations of one oxidative metabolite (MCOP) and the hydrolytic metabolite (MNP) of DINP, as well as MCNP, an oxidative metabolite of DIDP, in the general U.S. population. MCOP and MCNP were detectable in most persons examined, whereas MNP was detected in only about 12%. These NHANES data suggest that the U.S. general population is often exposed to DIDP and DINP and highlights the need for additional studies to identify potential sources of DINP and DIDP. Of interest, the significantly higher frequency of detection and urinary concentrations of MCOP than of MNP confirm the validity of MCOP as a biomarker for DINP exposure assessment and suggests widespread exposure to DINP. More important, these NHANES data suggest that the occurrence of exposure to DINP has been underestimated by using MNP as the sole DINP urinary biomarker.

## Figures and Tables

**Table 1:** 2 × 2 table for MCOP and MNP [unweighted no. of participants (weighted percent)].

	MNP urinary concentrations		
MCOP urinary concentrations	Detectable	Nondetectable	Total
Detectable	347 (12.9)	2 (0.02)	349 (12.9)
Nondetectable	2,100 (82.4)	99 (4.7)	2,199 (87.1)
Total	2,447 (95)	101 (5)	

**Table 2:** GM (95% CI) and selected percentiles of MCOP concentrations in urine for the U.S. population ≥ 6 years of age: data from NHANES 2005–2006.^a^

		Percentile (95% CI)						
	GM (95% CI)	10th	25th	50th	75th	90th	95th	*n*
Overall population								

μg/L	5.39 (4.68–6.22)	1.20 (1.00–1.40)	2.40 (2.10–2.80)	5.10 (4.40–6.00)	10.9 (9.10–13.1)	25.5 (19.1–35.4)	54.4 (32.5–85.2)	2,548
μg/g creatinine	5.26 (4.54–6.10)	1.53 (1.30–1.72)	2.60 (2.28–2.92)	4.53 (3.95–5.11)	9.25 (7.64–11.2)	24.0 (17.1–30.8)	40.2 (30.3–55.2)	2,548

Age group								

6–11 years								
μg/L	8.52 (7.19–10.1)	2.50 (1.90–3.40)	4.80 (4.00–5.80)	8.90 (7.60–9.90)	15.0 (11.3–19.3)	26.4 (19.9–37.3)	40.3 (26.4–61.6)	356
μg/g creatinine	9.38 (8.08–10.9)	3.29 (2.99–4.26)	5.53 (4.77–6.39)	8.49 (7.66–10.1)	14.8 (12.2–18.7)	30.3 (20.0–38.8)	40.1 (28.2–57.3)	356

12–19 years								
μg/L	6.61 (5.42–8.06)	1.60 (1.20–2.00)	3.40 (2.80–3.90)	6.30 (5.20–7.30)	11.9 (9.60–16.0)	28.4 (18.2–58.7)	63.2 (28.4–106)	702
μg/g creatinine	4.93 (4.10–5.93)	1.73 (1.34–1.98)	2.62 (2.26–3.02)	4.32 (3.61–4.89)	7.44 (5.93–11.0)	19.4 (12.4–31.8)	34.7 (24.1–58.3)	702

20–59 years								
μg/L	5.19 (4.40–6.12)	1.10 (0.800–1.50)	2.20 (1.80–2.60)	4.70 (4.00–5.70)	10.7 (8.50–13.1)	26.0 (17.9–44.6)	64.8 (32.6–99.9)	1,040
μg/g creatinine	4.98 (4.22–5.87)	1.43 (1.21–1.69)	2.42 (2.11–2.78)	4.10 (3.66–4.63)	8.59 (7.05–10.9)	24.8 (15.3–35.4)	43.7 (32.2–69.8)	1,040

≥ 60 years								
μg/L	4.25 (3.60–5.02)	1.00 (0.700–1.30)	2.10 (1.60–2.50)	3.70 (3.00–4.80)	8.20 (6.90–10.7)	20.1 (13.6–28.1)	37.0 (24.8–63.2) 450	
μg/g creatinine	4.95 (4.11–5.95)	1.48 (1.14–1.81)	2.48 (1.82–3.13)	4.46 (3.64–5.61)	8.48 (7.29–10.3)	18.5 (14.7–25.2)	30.9 (24.3–50.5)	450

Sex								

Male								
μg/L	6.24 (5.25–7.42)	1.50 (1.30–1.60)	2.80 (2.40–3.20)	5.70 (4.70–6.90)	12.1 (10.2–15.1)	30.8 (19.0–58.8)	66.4 (33.8–122)	1,270
μg/g creatinine	5.01 (4.21–5.97)	1.40 (1.21–1.63)	2.32 (2.04–2.68)	4.29 (3.75–5.03)	8.44 (7.17–10.9)	21.6 (15.1–35.4)	44.6 (25.7–85.9)	1,270

Female								
μg/L	4.69 (4.14–5.30)	1.00 (0.700–1.30)	2.10 (1.80–2.40)	4.50 (4.00–5.20)	9.30 (8.20–11.1)	21.5 (17.5–27.0)	41.7 (28.1–61.7)	1,278
μg/g creatinine	5.51 (4.75–6.39)	1.69 (1.39–2.08)	2.82 (2.56–3.17)	4.73 (4.23–5.33)	9.55 (8.26–12.2)	24.7 (17.3–30.3)	39.3 (30.3–45.0)	1,278

Race/ethnicity								

Non-Hispanic white								
μg/L	5.25 (4.40–6.27)	1.10 (0.900–1.40)	2.40 (2.00–2.70)	5.00 (4.10–6.20)	11.1 (8.60–13.6)	25.5 (17.7–43.5)	56.1 (28.1–88.5)	1,038
μg/g creatinine	5.53 (4.61–6.63)	1.60 (1.32–1.96)	2.78 (2.44–3.06)	4.81 (4.13–5.56)	10.0 (7.63–13.3)	25.2 (16.3–34.5)	39.3 (26.8–59.7)	1,038

Mexican American								
μg/L	5.77 (4.84–6.87)	1.40 (1.20–1.60)	2.40 (2.10–2.90)	5.20 (4.50–5.70)	9.90 (8.50–12.0)	23.6 (14.9–49.7)	54.4 (23.0–185)	637
μg/g creatinine	5.19 (4.38–6.14)	1.49 (1.33–1.84)	2.52 (2.28–2.73)	4.50 (3.64–5.29)	8.04 (7.13–10.1)	19.5 (12.3–37.7)	43.6 (20.9–93.4)	637

Non-Hispanic black								
μg/L	6.08 (5.60–6.61)	1.60 (1.40–1.80)	2.90 (2.60–3.30)	6.00 (5.10–6.70)	11.4 (10.4–13.0)	24.5 (20.3–28.1)	50.0 (32.0–70.3)	678
μg/g creatinine	4.28 (3.86–4.74)	1.40 (1.16–1.64)	2.14 (1.90–2.26)	3.67 (3.26–4.09)	7.60 (6.80–8.81)	17.1 (13.2–21.5)	31.8 (22.5–43.7)	678

a Participants not defined by the three racial/ethnic groups shown were included only in the total population estimate. LOD = 0.7 μg/L.

**Table 3:** GM (95% CI) and selected percentiles of MCNP concentrations in urine for the U.S. population ≥ 6 years of age: data from NHANES 2005–2006.^a^

		Percentile (95% CI)						
	GM (95% CI)	10th	25th	50th	75th	90th	95th	*n*
Overall population								

μg/L	2.73 (2.50–2.98)	< LOD	1.40 (1.20–1.50)	2.70 (2.40–3.00)	5.30 (4.80–5.90)	10.2 (8.80–11.9)	17.5 (14.0–21.4)	2,548
μg/g creatinine	2.66 (2.43–2.91)	< LOD	1.47 (1.32–1.61)	2.48 (2.29–2.71)	4.48 (4.05–4.85)	8.67 (7.17–9.71)	13.2 (10.8–17.5)	2,548

Age group								

6–11 years								
μg/L	4.54 (3.94–5.24)	1.50 (0.90–2.00)	2.50 (2.20–3.10)	4.70 (4.00–5.60)	8.10 (6.80–9.60)	14.6 (10.8–19.1)	22.8 (16.7–35.3)	356
μg/g creatinine	5.00 (4.45–5.63)	1.67 (1.22–2.26)	3.01 (2.57–3.33)	5.06 (4.18–5.76)	7.48 (6.71–8.84)	16.1 (12.5–18.9)	23.5 (16.1–31.7)	356

12–19 years								
μg/L	3.18 (2.74–3.68)	0.700 (< LOD–1.10)	1.80 (1.50–2.10)	3.30 (2.90–3.80)	6.00 (4.70–7.20)	10.3 (7.60–13.7)	16.5 (11.5–21.5)	702
μg/g creatinine	2.37 (2.04–2.75)	0.894 (< LOD–1.09)	1.51 (1.23–1.72)	2.18 (1.91–2.52)	3.64 (2.94–4.55)	6.26 (5.15–8.70)	10.7 (7.57–14.1)	702

20–59 years								
μg/L	2.52 (2.22–2.85)	< LOD	1.20 (1.10–1.40)	2.50 (2.20–2.80)	4.90 (4.20–5.70)	9.40 (7.60–12.6)	17.0 (12.6–24.2)	1,040
μg/g creatinine	2.42 (2.17–2.69)	< LOD	1.34 (1.20–1.50)	2.21 (2.05–2.48)	3.95 (3.59–4.53)	7.46 (6.00–9.41)	11.2 (9.26–19.0)	1,040

≥ 60 years								
μg/L	2.51 (2.15–2.92)	0.700 (< LOD–0.80)	1.10 (0.90–1.40)	2.40 (1.90–2.70)	4.70 (3.90–5.80)	10.1 (7.40–12.5)	14.7 (12.1–26.3)	450
μg/g creatinine	2.92 (2.46–3.46)	0.913 (< LOD–1.11)	1.58 (1.35–1.88)	2.72 (2.41–3.03)	4.60 (3.72–5.22)	9.40 (6.43–12.5)	14.6 (11.2–19.2)	450

Sex								

Male								

μg/L	3.16 (2.82–3.54)	0.800 (< LOD–1.00)	1.70 (1.40–1.90)	3.00 (2.70–3.50)	5.90 (5.10–6.90)	11.0 (8.80–14.0)	19.5 (14.0–28.0)	1,270
μg/g creatinine	2.53 (2.28–2.82)	0.894 (< LOD–1.02)	1.40 (1.27–1.54)	2.36 (2.14–2.56)	4.38 (3.74–5.04)	7.42 (6.47–8.88)	13.0 (9.40–19.5)	1,270

Female								
μg/L	2.37 (2.13–2.65)	< LOD	1.10 (1.00–1.30)	2.30 (2.00–2.70)	4.80 (4.30–5.30)	9.30 (7.60–11.5)	14.7 (13.1–18.2)	1,278
μg/g creatinine	2.79 (2.47–3.15)	< LOD	1.52 (1.30–1.77)	2.65 (2.34–2.96)	4.51 (4.03–5.16)	9.41 (6.82–11.6)	13.5 (10.7–19.7)	1,278

Race/ethnicity								

Non-Hispanic white								
μg/L	2.67 (2.39–2.98)	< LOD	1.30 (1.10–1.50)	2.60 (2.30–2.90)	5.30 (4.70–6.10)	10.1 (8.20–12.6)	17.6 (12.6–24.3)	1,038
μg/g creatinine	2.81 (2.51–3.14)	< LOD	1.55 (1.36–1.75)	2.57 (2.34–2.85)	4.62 (4.10–5.19)	9.26 (7.00–10.8)	13.5 (10.3–23.5)	1,038

Mexican American								
μg/L	2.72 (2.40–3.08)	0.700 (< LOD–0.90)	1.50 (1.20–1.80)	2.70 (2.40–3.20)	4.90 (4.30–5.70)	9.00 (7.00–12.4)	14.4 (9.00–26.8)	637
μg/g creatinine	2.45 (2.13–2.81)	0.824 (< LOD–1.12)	1.38 (1.26–1.53)	2.32 (2.14–2.60)	4.04 (3.64–4.58)	6.97 (5.73–8.14)	11.0 (7.89–19.1)	637

Non-Hispanic black								
μg/L	3.18 (2.75–3.67)	0.700 (< LOD–1.10)	1.60 (1.30–2.00)	3.20 (2.70–3.80)	5.90 (4.90–7.30)	13.0 (9.50–14.6)	19.2 (14.6–30.5)	678
μg/g creatinine	2.24 (1.96–2.55)	0.752 (< LOD–0.925)	1.26 (1.06–1.41)	1.97 (1.78–2.37)	3.60 (3.25–4.24)	7.82 (6.38–10.4)	12.0 (10.1–17.4)	678

a Participants not defined by the three racial/ethnic groups shown were included only in the total population estimate. LOD = 0.6 μg/L.

**Table 4:** Adjusted GM concentrations (95% CI) of MCOP and MCNP in various demographic groups.

Variable	Sample size (*n*)	GM (95% CI)	
		MCOP (μg/L)	MCNP (μg/L)
Household income			

< $20,000	576	4.62 (3.94–5.40)	2.35 (2.09–2.64)
$20,000–$45,000	782	5.07 (4.36–5.90)	2.65 (2.48–2.84)
> $45,000	1,118	5.86 (4.85–7.09)	2.88 (2.8–3.23)

Age group			

6–11 years	350	9.51 (8.19–11.03)	5.05 (4.52–5.64)
12–19 years	688	5.26 (4.36–6.34)	2.59 (2.25–2.97)
20–59 years	1,012	5.12 (4.37–5.99)	2.47 (2.23–2.73)
≥ 60 years	426	5.05 (4.24–6.01)	2.90 (2.45–3.44)

For both phthalate metabolites, adjusted GM concentrations for children 6–11 years of age were significantly higher (*p* < 0.01) than for all other age groups. Similarly, adjusted GM concentrations for persons in the high household income category were significantly higher than for those in the low category (*p* = 0.004 for MCNP; *p* =0.01 for MCOP); for MCNP, persons in the medium income level had significantly higher adjusted GM concentrations than those in the low income level (*p* = 0.03). The final MCOP and MCNP models included household income, age group, and log_10_ creatinine; covariates data were missing for 72 participants.
